# Central sensitization in adolescents with hypermobility spectrum disorder or hypermobile Ehlers-Danlos syndrome—a feasibility study

**DOI:** 10.1186/s40814-023-01320-3

**Published:** 2023-06-14

**Authors:** Elke Schubert-Hjalmarsson, Anders Fasth, Kelly Ickmans, Eva-Lott Mårdbrink, Ann-Charlott Söderpalm, Mari Lundberg

**Affiliations:** 1 Division of Physiotherapy, Department of Health and Rehabilitation, Institute of Neuroscience and Physiology, Sahlgrenska Academy, University of Gothenburg, Gothenburg, Sweden; 2 Department of Physiotherapy, Queen Silvia Children’s Hospital, Sahlgrenska University Hospital, Region Västra Götaland, Gothenburg, Sweden; 3 Department of Pediatrics, Institute of Clinical Sciences, Sahlgrenska Academy, University of Gothenburg, Gothenburg, Sweden; 4 Department of Rheumatology and Immunology, Silvia Children’s Hospital, Sahlgrenska University Hospital, Region Västra Götaland, Gothenburg, Queen Sweden; 5 Pain in Motion (PAIN) Research Group, Department of Physiotherapy, Human Physiology and Anatomy, Faculty of Physical Education & Physiotherapy, Vrije Universiteit Brussel, Brussels, Belgium; 6 Department of Physical Medicine and Physiotherapy, University Hospital Brussels, Brussels, Belgium; 7 Movement & Nutrition for Health & Performance Research Group, Department of Movement & Sport Sciences, Faculty of Physical Education & Physiotherapy, Vrije Universiteit Brussel, Brussels, Belgium; 8 Department of Orthopaedics, Institute for Clinical Sciences, Sahlgrenska Academy, University of Gothenburg, Gothenburg, Sweden; 9 Department of Health Promoting Science, Sophiahemmet University, Stockholm, Sweden; 10 University of Gothenburg Centre for Person-Centred Care (GPCC), Sahlgrenska Academy, University of Gothenburg, Gothenburg, Sweden

**Keywords:** Feasibility study, Hypermobility, Ehlers-Danlos syndrome, Central sensitization, Pain

## Abstract

**Background:**

Pain is a major symptom in adolescents with hypermobility spectrum disorder or hypermobile Ehlers-Danlos syndrome. Although the underlying mechanism causing generalized pain in children with hypermobility spectrum disorder or hypermobile Ehlers-Danlos syndrome is unclear, central sensitization has been suggested as a possible explanation. The aim of this study was to explore the feasibility of a study protocol for a future case–control study, investigating features of central sensitization in adolescents with hypermobility spectrum disorder or hypermobile Ehlers-Danlos syndrome.

**Methods:**

Central sensitization features were measured in ten patients and nine healthy controls aged 13–17 years via experimental pain measurement quantifying primary and secondary hyperalgesia, endogenous pain modulation, and exercise-induced hyperalgesia. Descriptive statistics were used. Frequency, median, and range values were calculated.

**Results:**

Eleven out of 57 patients chose to participate. No control could be recruited through public schools. Therefore, a convenience sampling strategy was used for the recruitment of the control group. The process of assessing primary and secondary hyperalgesia, endogenous pain modulation, and exercise-induced hyperalgesia was well tolerated by all participants (patients and controls). When assessing endogenous pain modulation via conditioned pain modulation, two participants in the patient group and three in the control group did not achieve a pain experience ≥ 3 on the numerical rating scale when immersing their hands in cold water.

**Conclusion:**

This study investigated the feasibility, safety, and toleration of experimental pain measurements in adolescents with hypermobility spectrum disorder or hypermobile Ehlers-Danlos syndrome. Although the test protocol proved to be sufficiently feasible for use with the participant group, it will need to be adapted in the main study in order to obtain more reliable data. Recruitment, especially of participants for the control group, can be a major obstacle for future studies and requires careful planning.

**Trial registration:**

Researchweb.org, 270,501. Registered on 9 May 2019.

## Key messages regarding feasibility

- There is a need to increase knowledge about underlying factors for pain in adolescents with HSD/hEDS.
- This study demonstrates the feasibility of the tested protocol regarding safety, feasibility, and tolerance of the measurement methods in adolescents.
- The study showed that the protocol will require some adjustment to ensure that its measurements generate reliable results, such as reducing the water temperature to ensure a painful stimulus.
- Future studies should aim to improve and extend the recruitment process by including more information channels, such as social media and patient associations.

## Background

Hypermobility spectrum disorder (HSD) and hypermobile Ehlers-Danlos syndrome (hEDS) are two heritable connective tissue disorders characterized by generalized joint hypermobility, chronic and progressive pain, and fatigue [1, 2]. While the prevalence of hEDS is reported as 1:10,000–15,000 [3], no separate data are available for the paediatric population. Adolescents with HSD/hEDS participate less often in social activities and have a lower self-reported and health-related quality of life compared with their healthy peers [4, 5]. The mechanisms underlying HSD/hEDS are poorly understood, making their assessment and management uncertain, particularly in adolescents [1].

Although the underlying mechanism causing generalized pain in children with HSD/hEDS is unclear, central sensitization (CS) has been suggested as a possible explanation [6]. CS is an umbrella term covering several central pain-enhancing mechanisms and is defined as *an amplification of neural signalling within the central nervous system that elicits pain hypersensitivity* [7]. Clinical signs of CS include hypersensitivity to pain, but also to other sensory stimuli such as touch, smell, sound, and light, as well as widespread pain outside the primary area of tissue injury/damage [8]. The current accumulated body of scientific knowledge regarding CS has resulted in a shift from primarily considering peripheral mechanisms in patient management to considering central mechanisms [9].

CS can occur as a result of either acute and/or prolonged peripheral pain triggers and can include disturbances in both the ascending and descending nervous systems. Due to this complexity, it is considered most reliable to use several measurement methods simultaneously to detect CS [10]. Hence, in this study, three measurement methods were applied: (1) primary and secondary hyperalgesia, which investigates increased sensitivity to pain/discomfort [11]; (2) endogenous pain modulation, which investigates whether the pain experience changes when the subject is exposed to a second painful stimulus [12, 13]; and (3) exercise-induced hypoalgesia, which investigates the attenuation of pain following single episodes of exercise [14]. To date, generalized hyperalgesia measured using primary and secondary hyperalgesia has been noted in adolescents with HDS/hEDS [15]. On the other hand, endogenous pain modulation and exercise-induced hyperalgesia have never been investigated in this population. These measurement methods for assessing features of CS will be tested in the present feasibility study. The results will be used to improve the study protocol for larger future case control study.

The aim of this study was to investigate the feasibility of using certain measurement methods for assessing features of CS in adolescents with HSD/hEDS in comparison with control group subjects with no known ailments.

### Ethical considerations

This study was approved by the Swedish Ethical Review Authority (ref. no. 2019–03446).

## Methods

### Design

This study was designed so as to determine the feasibility of a future experimental case–control study.

### Recruitment

The study sample consisted of one patient group and one control group.

All patients at Queen Silvia Children’s Hospital in Gothenburg, Sweden, diagnosed with HSD/hEDS aged 13–17 years were identified via the electronic medical records, using the search terms ‘ICD 10 Q769’ and ‘M357’. The control group was recruited via schools in the region. Participants in the control group were matched for gender and age.

The exclusion criteria for both groups were as follows: (1) not being able to read or understand Swedish, (2) being pregnant or 1 year postpartum, and (3) having a (co-morbid) neurological, rheumatic, musculoskeletal, or metabolic disorder. Participants in the control group must not have experienced pain that persisted for more than 24 h during the 3 months prior to their examination. In the patient group, participants were excluded if they had other syndromes including hypermobility, such as Marfan syndrome, osteogenesis imperfecta, or other types of EDS. Participants in the control group needed to lead a less active lifestyle overall, and subjects were therefore excluded if they exercised more than three times per week.

A preliminary review of the patients’ medical records was conducted to confirm their diagnosis and to ensure that they fulfilled the inclusion criteria.

### Procedure

The adolescents in both groups and their parents/guardians were informed about the study by letter and asked to participate. In this letter, control group participants were informed that they needed to be healthy and pain-free in order to participate. All participants and their parents/guardians were given the opportunity to receive further information orally. Participants under 15 years of age gave verbal assent, and participants over 15 years of age as well as all guardians provided written informed consent before being invited to visit the clinic.

All participants—both patients and control subjects—and their guardians visited the hospital on two occasions, 7 days apart. On the first occasion, the participants underwent a medical examination by an independent physician in line with the 2017 criteria for HSD/hEDS [16, 17]. This was to verify that the selected patients fulfilled the 2017 criteria, and that the control group participants did not. Thereafter, an independent observer asked all participants to fill in the self-reported questionnaire. In addition, participants were asked not to do any physical exercise at least 48 h before the measurements and to refrain from consuming caffeine, alcohol, and nicotine on the day of the second measurements. For ethical reasons, patients were allowed to take non-opioid pain medication as described in the first step of the World Health Organization analgesic ladder (COX inhibitors and paracetamol). However, we asked them to abstain from taking these medications for 24 h prior to their second measurements.

On the second assessment day, all participants returned to undergo the CS measurements. An independent investigator informed the children about how the measurements were to be carried out. Instrument placement points were measured out and marked with a pencil on the participants’ skin. Pressure pain threshold (PPT) measurements were repeated three times at each measurement point. During a 10-min break, the participants washed their hands and were offered juice to drink. Next, the participants placed their non-dominant hand in the cold water, and for the conditioned pain modulation, the PPT measurements on the trapezius muscle were repeated. Self-reported and experimental pain measurements were recorded both during and immediately after the participants removed their hands from the water. Following a 15-min break, participants performed a submaximal workout on a bicycle ergometer. The self-reported and experimental pain measurements as well as self-estimated exhaustion were recorded immediately after participants completed this exercise.

The first author was responsible for conducting recruitment, enrolment, and allocation to the intervention for all participants. The independent physician, the independent observer, and the independent investigator were blinded to the group affiliation.

### Assessments

**Fig. 1:**
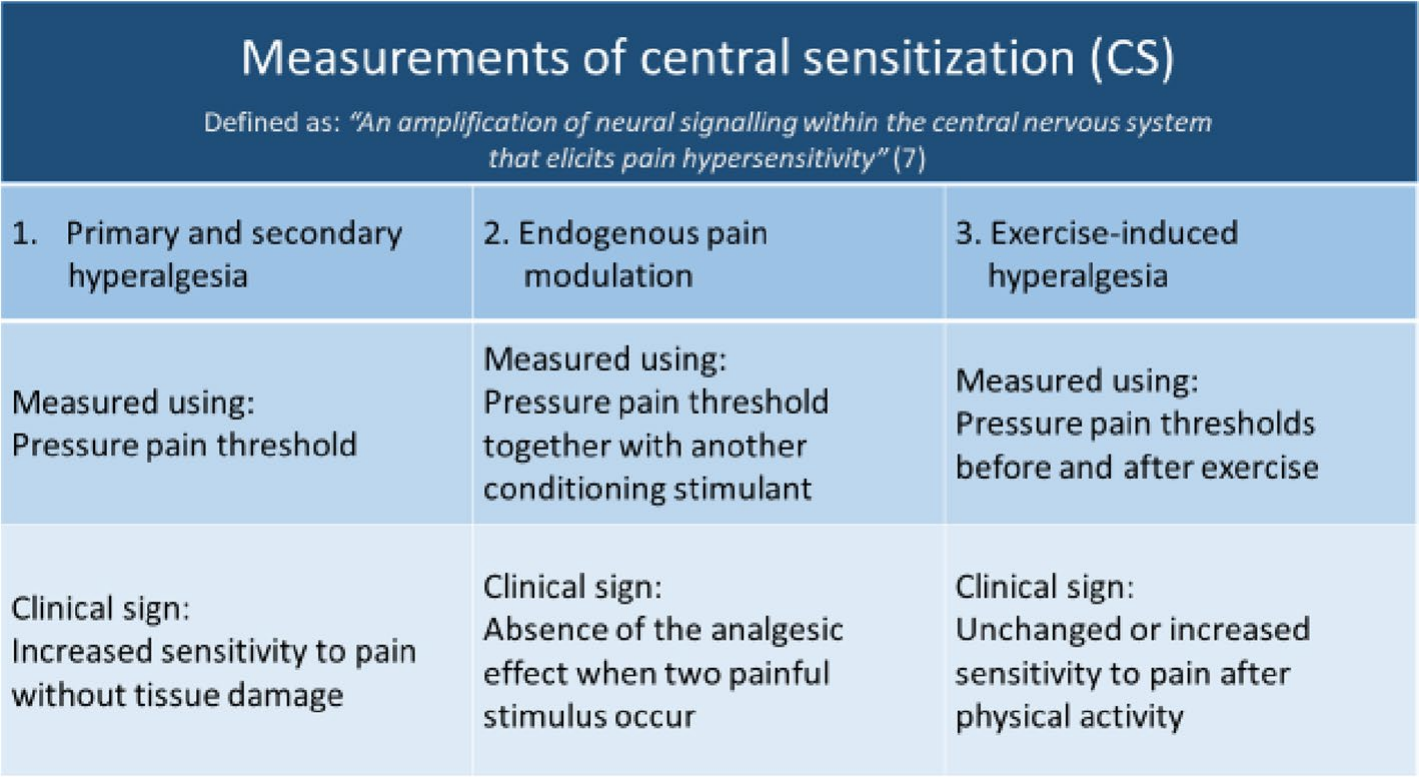
Presentation of the measurement methods used to investigate the features of central sensitization (CS) in this study

For the purpose of this feasibility study, CS features were assessed using three different measurement methods: (1) primary and secondary hyperalgesia, (2) conditioned pain modulation, and (3) exercise-induced hyperalgesia (Fig. 1).

#### Primary hyperalgesia and secondary hyperalgesia

 Were assessed by measuring PPTs at a symptomatic test site (trapezius muscle) and three remote test sites (triceps muscle, lower back, and tibialis anterior muscle (upper and lower quadrant and axial)) using a hand-held pressure algometer (Wagner Instruments, FPX 25) [18]. The algometer’s 1-cm^2^ rubber tip was positioned at an angle of 90° relative to the body part being assessed, and pressure was applied at a rate of 1 kg/s. The participants laid down in a prone position when measuring the PPTs on their lower back, laid down in a supine position when measuring the PPTs on their triceps and the tibialis anterior test sites, and remained seated while measuring at the trapezius test site. They were instructed to say ‘stop’ as soon as the pressure sensation changed to one of discomfort. At that point, the pressure algometer was removed and the value on the display was recorded. This procedure was repeated three times at each test site (30 s between each measurement). The PPT was established as the mean of the last two values, since this approach has been found to be reliable in both children and adults [18].

#### Endogenous pain modulation

 Was assessed via conditioned pain modulation (CPM). Within the CPM paradigm, the perceived pain intensity of a test stimulus is measured before, during, and after the addition of a painful conditioning stimulus. In this study, the cold pressure task was used as a ‘conditioning stimulus’ and a mechanical task as a ‘test stimulus’.

*Test stimulus*: The PPT at the trapezius test site served as a test stimulus. As mentioned in the section above, the PPT was measured using a hand-held pressure algometer (Wagner Instruments, FPX 25) with a 1-cm^2^ rubber tip and using an application rate of 1 kg/s.

*Conditioning stimulus*: Participants were asked to place their non-dominant hand in a cold pressor unit (Fisher Scientific, Thermo Scientific™ 231,131,300, VersaCool™ Refrigerated Circulating Bath) with a constant water temperature of 12 °C ± 1 [19]. To prevent localized warming around the hand, the water was kept in motion via a water circulator. Participants were instructed to leave their hands in the water for 1 min until they were told to take it out. Immediately after removing their hand from the water, participants were asked to rate the pain caused by the immersion using the Numerical Rating Scale (NRS, as explained below).

*Procedure*: The test stimulus (PPT trapezius) was administered first without the cold water conditioning stimulus. Next, the cold pressure task was initiated, and the participants’ non-dominant hand was immersed in cold water. After 20 s and 50 s of immersion, respectively a test stimulus (PPT trapezius) was administered for a second and a third time.

The outcome measure for CPM was obtained by subtracting the mean PPT recorded during the conditioning stimulus from the mean PPT recorded prior to the conditioning stimulus. Thus, a positive value represents a decrease in the patient’s pain threshold, reflecting pain facilitation or abnormal CPM, while a negative value represents pain inhibition or normal CPM [20].

#### Exercise-induced hypoalgesia (EIH)

Was assessed by repeating the PPT measures described above immediately after performing an exercise test on a bicycle ergometer, as described by Furzer et al. [21]. To induce exertion rather than exhaustion in participants, their cycling exercise was interrupted when they reached 75 of their expected maximum heart rate (i.e., 220 beats per minute minus their age in years) as the target value, or when they reported a perceived exertion rating of 15 on the Borg Rating of Perceived Exertion Scale (Borg RPE) 6–20, with 6 = *no exertion* and 20 = *maximal exertion* [22]. Maximal exercise capacity values were recorded, including peak heart rate during a cycling test. Self-estimated exhaustion was also measured using the Borg RPE scale. It was also noted whether the child was interrupted due to either fatigue in their legs or cardiovascular exhaustion.

The outcome measure for EIH was obtained by subtracting the mean PPT after the exercise from the mean PPT prior to the exercise. A positive value represents a decrease in pain threshold, reflecting pain facilitation or impaired EIH, while a negative value represents pain inhibition or normal EIH.

The testing order of the PPT measurement points during the PPT evaluation and the EIH measurements was randomized for each group. To generate the randomization list, www.jerrydallal.com was used [23].

The average pain intensity caused by immersion in cold water was measured using a Numerical Rating Scale (NRS) of 0–10 [24], with 0 = *no pain* and 10 = *worst possible pain* as the endpoints. The NRS-11 is commonly used to assess pain intensity in pain research conducted on young people [24–26], and there is good evidence to support its reliability and validity in individuals as young as 6 years of age [26, 27].

The same room and bicycle were used in order to standardize the environment for each of the assessments. All measurements were recorded in the afternoon and at the end of the week.

### Data analysis

The purpose of this feasibility study was to describe the group. Thus, only descriptive statistics were used. Frequency, median, and range values were calculated (IBM SPSS Statistic 26).

## Results

### Patient recruitment

**Fig. 2:**
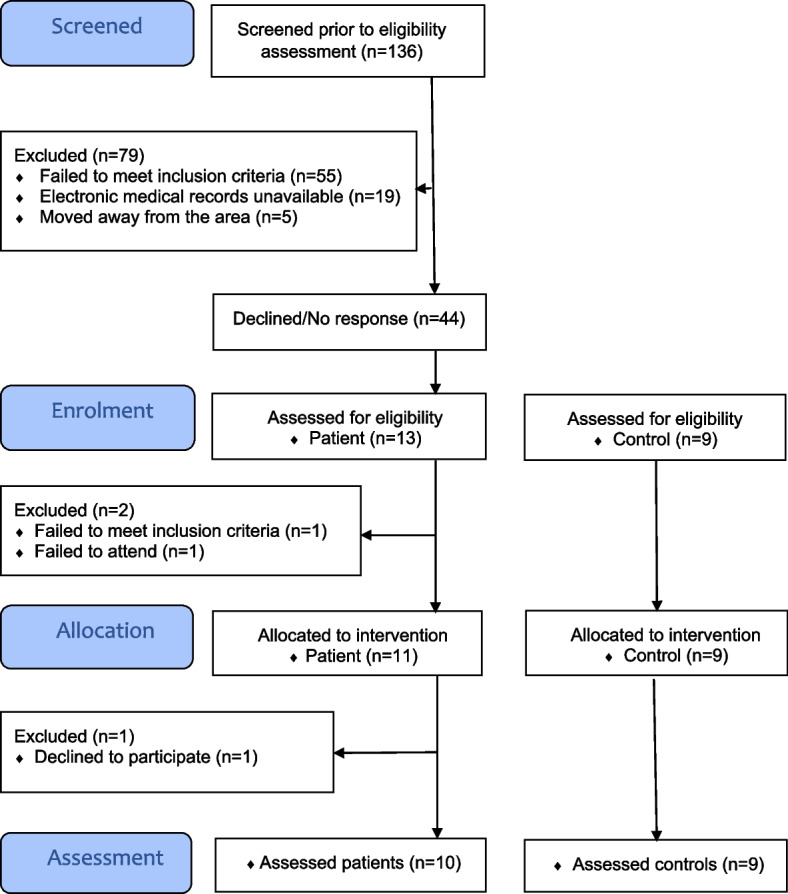
Participant flow

One hundred thirty-six patients were identified. After reviewing their medical records, it was found that 55 of these patients did not satisfy the inclusion criteria. Electronic medical records were not available for a further 19 patients, and five patients had moved away from the area. The remaining 57 patients were invited to participate via mail. After 23 days, nine patients had agreed to participate, ten had declined, and 38 had not responded. After 1 month, a reminder was sent to the 38 patients who did not respond initially. As a result, four more candidates agreed to participate, one declined, and 33 did not respond. Over a period of 2 months, 13 patients agreed to participate, of whom three later were excluded. One was because of another motor impairment disorder that was overlooked in the medical records review, one declined, and the third failed to attend (Fig. 2).

### Control group recruitment

Once the patients had agreed to participate, the researchers began recruiting control subjects, contacting six schools in the Gothenburg area. Two schools responded and allowed the researchers to contact their school nurse for assistance with recruitment, two schools declined, and two did not respond. No single healthy control was recruited by these means. The researchers then turned to friends and colleagues, a so-called convenience sampling strategy, and were able to recruit nine healthy controls. When asked about their participation, the participants in the control group reported that they considered themselves to be both healthy and pain-free.

The characteristics of the two groups are described in Table 1.

**Table 1:** Description of the study population

	Patient group (*n* = 10), median (range)	Control group (*n* = 9), median (range)
Age (years)	15 (13 to 17)	15 (14 to 17)
Gender	4 boys, 6 girls	4 boys, 5 girls
Beighton score (0–9)	4 (2 to 8)	4 (2 to 9)
BMI (kg/m^2^)	19 (17 to 29)	20 (19 to 24)
Reported occasions of planned physical activity/week	0.5 (0 to 3)	0 (0 to 2)

*BMI* body mass index

### Self-reported pain

Median pain intensity over the past week as rated using the NRS was reported as 3.5 (range: 0–8) for the patient group and 0 (range: 0–3) for the control group. Median pain intensity at the time of the assessment was reported as 1.5 (range: 0–5) for the patient group and 0 (range: 0) for the control group (Table 2). Seven participants in the patient group reported that their pain affected them to such an extent that they were unable to participate in all aspects of their education. The remaining participants in both groups did not experience any impairments in connection with their schooling.

**Table 2:** Pain demographics

	Patient group (*n* = 10)	Control group (*n* = 9)
**Pain intensity (NRS)**	Median (range)	Median (range)
Over the past week	3.5 (0 to 8)	0 (0 to 3)
At the time of the assessment	1.5 (0 to 5)	0 (0)
**Pain duration**	Frequency	Frequency
No pain	0	4
Less than 3 months	1	1
1.5 years	2	2
More than 5 years	7	2
**Pain localization**	Frequency	Frequency
Legs (unspecified)	5	0
Hips	4	0
Knees	5	3
Feet/toes	9	1
Shoulder/neck	7	1
Hands/wrists	3	1
Back/torso	3	0
Stomach	3	1

*NRS* Numerical rating scale

When asked how often they experienced pain, three participants in the patient group reported feeling pain all the time, two felt pain several times per day, one several times per week, two once per week, one several times per month, and one reported feeling no pain at all. In the control group, three participants reported experiencing pain once per week, and two others once per month. The duration of participants’ pain varied between upwards of 3 months and more than 5 years (Table 2).

A total of one participant in the patient group and seven in the control group did not experience any limitations due to pain. Four participants in the patient group experienced minor limitations, three experienced moderate limitations, and one experienced major limitations. Two participants in the control group experienced minor limitations due to pain (Table 2).

All participants within the patient group reported experiencing pain in several body parts. In the control group, five participants reported one localization and one participant reported experiencing pain in multiple body parts. Pain in the lower extremity was most frequently reported, with all ten patients reporting pain. Five participants in the control group reported some form of pain localization. One participant reported feeling pain in multiple body parts (knees, toes, and hands) and the remaining four participants in one body part (Table 2).

Seven participants in the patient group and two participants in the control group related that they had a family member who had suffered pain for an extended period.

### Feasibility of measuring central sensitization

The primary and secondary hyperalgesia, endogenous pain modulation, and exercise-induced hypoalgesia assessments were all well tolerated by the participants. All assessments were performed according to plan. No adverse reactions to the measurement methods were reported. The instructions were clear to the participants.

When assessing CPM, two participants in the patient group and three participants in the control group did not achieve a pain experience (NRS ≥ 3) when immersing their hands in the cold water. Two participants in the patient group and three in the control group tolerated less pressure during CPM assessments. The other participants tolerated greater pressure.

Four participants in the patient group and four in the control group tolerated less pressure being applied to the trapezius muscle after cycling, and four participants in the patient group and five in the control group tolerated less pressure when measuring at the tibialis anterior muscle, indicating exercise-induced hyperalgesia (Table 3). One patient interrupted cycling on the ergometer bicycle due to fatigue in their legs. The other participants, nine patients and nine controls, cycled to an exertion level equal to 75% of their expected maximum heart rate. Pain in the knees and/or legs was reported by all patients (*n* = 10) and by five adolescents in the control group.

**Table 3:** Description of pain measurement outcomes, median/(range)

Measurements	Patient group (*n* = 10), median (range)	Control group (*n* = 9), median (range)
PPT—trapezius muscle (kg/cm^2^)	2.01 (1.52 to 5.04)	3.03 (1.3 to 5.05)
PPT—deltoideus muscle (kg/cm^2^)	1.94 (0.67 to 3.28)	1.8 (1.06 to 5.53)
PPT—tibialis anterior (kg/cm^2^)	3.02 (2.05 to 7.31)	4.53 (1.58 to 7.79)
PPT—lower back (L3) (kg/cm^2^)	2.85 (1.56 to 6.88)	3.71 (1.94 to 10.54)
CPM outcome (kg/cm^2^)	0.6 (− 0.23 to 1.23)	0.5 (− 0.82 to 1.33)
EIH outcome—trapezius muscle (kg/cm^2^)	0.05 (− 0.48 to 2.01)	0.27 (− 1.17 to 0.59)
EIH outcome—tibialis anterior (kg/cm^2^)	0.44 (− 1.3 to 2.91)	0.04 (− 1.51 to 1.01)
Borg RPE (6–20)	16.5 (15 to 19)	14 (13 to 15)
Cycling duration (min)	5 (4 to 6)	4 (3 to 5)
NRS—cold pressure test	5.45 (2.3 to 6.8)	1.0 (0 to 7.8)

*Borg RPE* Borg Rating of Perceived Exertion Scale, *CPM* Conditioned pain modulation, *EIH* Exercise-induced hypoalgesia, *L3* Lumbar vertebra no. 3, *NRS* Numerical Rating Scale, *PPT* Pressure pain threshold

## Discussion

It is not ethically defensible to conduct a randomized controlled trial without having tested the feasibility of the study protocol, especially when it involves inflicting pain on young participants. Thus, the purpose of this study was to investigate whether the proposed study protocol was safe and feasible in connection with adolescent subjects with HDS/hEDS or if adjustments were needed prior to conducting a larger study.

The study protocol was found to be safe inasmuch as it caused no side effects. On the other hand, the study yielded important insights concerning the feasibility and quality of the study protocol that merit further consideration. This study assessed the features of CS using a combination of three different measurement methods: primary and secondary hyperalgesia, conditioned pain modulation, and exercise-induced hyperalgesia*. Primary hyperalgesia and secondary hyperalgesia* were assessed by measuring PPTs at a symptomatic test site (trapezius muscle) and three remote test sites (triceps muscle, lower back, and tibialis anterior muscle (upper and lower quadrant and axial)). Based on the findings of a previous study performed on adolescents with pain [28], the trapezius muscle was assumed to be the primary pain localization in individuals with HSD/hEDS. However, the study population reported pain mostly in their lower extremities and, in only a few cases, in the shoulder area. Due to the natural history of pain in individuals with HSD/hEDS—in which prolonged musculoskeletal pain is one of the diagnostic criteria and generalized pain manifesting in different areas that may vary over time in the same individual are commonplace—it is difficult to define a primary symptomatic pain point. Consequently, using the term ‘generalized secondary hyperalgesia’ concerning the occurrence of hyperalgesia even in asymptomatic areas might be more accurate [6, 15]. The use of the term ‘generalized secondary hyperalgesia’ should be considered in any future study.

In the present study, *endogenous pain modulation* was assessed via CPM [29], with the participants immersing their hands in cold water as a way to trigger pain. In this paradigm, 12 °C was considered a sufficiently cold pain stimulus to activate the pain-regulating system [19]. However, in five of the participants (two patients and three controls) the cold water did not trigger any pain (NRS < 3), which raised the question of whether the selected temperature was too warm. The literature suggests that a mild to moderate level of pain (NRS 2/10) should be sufficient [30–32] for the conditioning stimulus to have an effect. When using cold water, it is important to select a temperature that allows a child to hold their hand in the water for 1 min while still being sufficiently cold to trigger a pain reaction. Previous studies have shown that about 50% of children pull their hands out of water chilled to 10 °C after 1 min [33]. This percentage increases to 60% when the temperature is set at 7 °C, and again to 70% when the water temperature is 5 °C [34]. A temperature of 12 °C was chosen for this study to ensure that participants were able to hold their hands in the water for 1 min. Unlike studies that measure pain tolerance, this study set a maximum time limit for the immersion, which might have motivated children to keep their hands in the water even when their discomfort was greater. Other studies have used water chilled to 10 °C as part of a cold pressure task designed for children [35]. As such, reducing the water temperature to 10 °C could be a good compromise. While it entails an increased risk that the children will not be able to keep their hands in the water, it also increases the likelihood that more participants will find the cold water sufficiently unpleasant to stimulate a pain response.

### Limitations in measuring CPM

We did not investigate whether external factors such as climate and season might cause the participants’ initial hand temperatures to vary. Beayer et al. recommend that participants first immerse their hands in water with a temperature of 35–37 °C for 2 min to mitigate any initial differences in skin temperature [35]. Doing so would also give participants the opportunity to get used to the examination environment, and the time spent could be used to explain the pain assessment to the child [35]. This study did not take this approach, however, and it is not possible to comment on whether doing so would have altered how participants experienced the cold water.

Another variable that the researchers did not take into consideration was the time required for each individual pain measurement examination. Thus, we are unable to report on this element even though we gained some sense of the time required while conducting the tests.

*Exercise-induced hypoalgesia (EIH)* was assessed by repeating the PPT measurements described above immediately after participants underwent an exercise test on a bicycle ergometer. When measuring EIH in this study, eight out of ten patients tolerated more pressure after exercise, which is consistent with EIH, while two tolerated less pressure after exercise. This indicates an altered pain response in these patients. In the control group, six of nine participants tolerated more pressure after exercise, which is consistent with EIH. Three patients tolerated less pressure after exercise, which indicates an altered pain response. In a pain-free individual, exercise should lead to a reduction in their pain experience [36].

### Limitations in measuring EIH

In order to investigate the occurrence of EIH using the current protocol, participants were asked to cycle for 4–5 min. Other study protocols have adopted a minimum duration of 8 min or more [36], which may be more appropriate. To enable participants to report any change in their pain sensitivity in response to physical activity, it is important that the intensity and duration of physical exercise tests be calibrated so as to cause exercise-induced hypoalgesia in healthy individuals [14]. Previous studies’ results regarding dose response for EIH vary. One study shows a dose response with greater effects after 30 min of cycling as compared with 10 min of cycling [37], while another study found no discernible difference between cycling for 10 min and 20 min, respectively [38]. Moreover, there are studies that show that very short-term aerobic exercise can induce EIH [39, 40]. Vaegter et al. conclude that ‘the combination of intensity and duration may be more important in determining the magnitude of the EIH after aerobic exercise than any of the variables alone’ [41]. With this in mind, it may be beneficial to review the exercise protocol in preparing for any future study. Additionally, some control participants involved in this study were found to have mild though persistent pain problems, which might also have caused a distorted result when measuring EIH.

This study measured EIH and CPM in close proximity. Previous studies have shown CPM to have no lasting effect [38, 42]. The minimum recommended interval between CPM and EIH measurements is 10 min [43, 44]. This study opted to set an interval of 15 min between CMP and EIH measurements. This circumstance lends support to the assumption that switching between these two measurements should not influence cycling’s hypoalgesic effect in a larger trial.

### Limitations affecting recruitment

Recruitment of participants for the control group via colleagues and friends, a convenience sampling strategy, may involve a risk that participants feel more compelled to participate in the study. There is also a risk of similar sociocultural environment, which can reduce comparability between the groups. However, the investigators were blinded to group affiliation, reducing the risk of bias.

One of this study’s inclusion criteria was that participants in the control group be pain-free. At the time of their recruitment, parents and adolescents were asked if they were suffering any pain before being invited to participate. However, upon more in-depth probing in connection with answering the questionnaire, some adolescents indicated that they suffered from some kind of pain. Two of them even experienced some limitations in daily life as a result of their pain. This circumstance serves to highlight the importance of asking more explicit and detailed questions regarding pain to the adolescents themselves before inviting them to participate in the study. On the other hand, since pain is a part of life, it cannot be completely ruled out that certain pain exists among participants when recruiting the control group. Some heterogeneity was also observed among the patient population in terms of the incidence and intensity of their pain. In any future study, it would be beneficial to improve the recruitment process in order to assemble groups that are better matched.

## Conclusion

The study protocol trialled in this investigation proved to be suitably feasible for use with the participant group, although it will require some adaptation in connection with the future main study in order to obtain more reliable data. It was challenging to recruit both patient and control participants. Recruitment, especially of the control group, can be a major obstacle for future studies and requires careful planning.

Based on the present study, in order to ensure a high-quality case–control study, the following changes are suggested: (1) reduce the water temperature from 12 to 10 °C to ensure a painful stimulus, (2) refine the ergometer cycling protocol by increasing the duration of physical activity tests, and (3) improve and extend the recruitment process by including more information channels, such as social media and patient associations.

## Data Availability

The datasets used and/or analysed as part of the current study are available from the corresponding author upon reasonable request.

## Abbreviations

CPM: Conditioned pain modulation
CS: Central sensitization
EIH: Exercise-induced hypoalgesia
EDS: Ehlers-Danlos syndrome
hEDS: Hypermobile Ehlers-Danlos syndrome
HSD: Hypermobility spectrum disorder
NRS: Numerical Rating Scale
PPT: Pressure pain threshold
Borg RPE: Borg Rating of Perceived Exertion Scale
